# Neurological Consequences of 2019-nCoV Infection: A Comprehensive Literature Review

**DOI:** 10.7759/cureus.8790

**Published:** 2020-06-24

**Authors:** Dua Azim, Sundus Nasim, Sohail Kumar, Azhar Hussain, Sundip Patel

**Affiliations:** 1 Internal Medicine, Dow University of Health Sciences, Karachi, PAK; 2 Internal Medicine, Dow Medical College and Dr. Ruth K. M. Pfau Civil Hospital, Karachi, PAK; 3 Healthcare Administration, Franklin University, Columbus, USA; 4 Medicine, Xavier University School of Medicine, Oranjestad, ABW; 5 Medicine, Windsor University School of Medicine, Cayon, KNA

**Keywords:** covid-19, sars-cov-2, hypoxic injury, immune-mediated injury, cerebrovascular injury

## Abstract

First identified in November 2019 in Hubei Province, the coronavirus disease of 2019 (COVID-19) caused by SARS-CoV-2 soon spread worldwide to become a global health pandemic. The COVID-19 preferentially damages the respiratory system that produces symptoms such as fever, cough, and shortness of breath. However, the infection often tends to disseminate to involve various organ systems. Recent evidence indicates that SARS-CoV-2 can cause significant neurological damage and resultant neurological symptoms and complications. Here, we provide a comprehensive and thorough review of original articles, case reports, and case series to delineate the possible mechanisms of nervous system invasion and damage by SARS-CoV-2 and subsequent consequences. We divided the neurological manifestations into three categories: (1) Central Nervous System involvement, (2) Peripheral Nervous System manifestations, and (3) Skeletal Muscle Injury. Headache and dizziness were found to be the most prevalent symptoms followed by impaired consciousness. Among the symptoms indicating peripheral nervous system invasion, anosmia and dysgeusia were commonly reported. Skeletal muscle injury predominantly presents as myalgia. In addition, encephalitis, myelitis, cerebrovascular disease, Guillain-Barre syndrome, and Miller Fischer syndrome were among the commonly noted complications. We also emphasized the association of pre-existing comorbidities with neurological manifestations. The aim of this review is to provide a deeper understanding of the potential neurological implications to help neurologists have a high index of clinical suspicion allowing them to manage the patient appropriately.

## Introduction and background

The first coronaviruses were identified in animals affecting the respiratory, gastrointestinal, and central nervous system (CNS) [1]. In the previous years, different researches have been done on the subject portraying the impacts of this virus on human health, especially in Asian and Middle East Countries. The zoonotic and human-to-human transmission of this virus was initially described in Guangdong City of China with the outbreak of the severe respiratory syndrome named Severe Acute Respiratory Syndrome-Coronavirus (SARS-CoV) which infected 8098 individuals with a mortality rate of 9%, across 26 countries in the world [2].

Nowadays, a highly contagious pandemic prevails, becoming the focus of concern for health care workers around the globe. This newly emergent coronavirus disease 2019 (COVID-19), first recognized in the city of Wuhan, Hubei Province of China in early December 2019, presents as an unusual case of pneumonia of unknown etiology [3]. The symptoms of COVID-19 resemble those of SARS-CoV of 2003. A logical explanation of the similarity of symptoms can be attributed to both viruses utilizing the same receptor, angiotensin-converting enzyme-2 (ACE2) [4]. With nearly two dozen countries affected, the outbreak of COVID-19 has deemed a public health emergency of global concern by the World Health Organization (WHO) on January 30, 2020. Furthermore, on February 11, 2020, the Coronavirus Study Group of the International Committee on Taxonomy of Viruses named the new virus ‘SARS-CoV-2’ due to its striking similarities to previous SARS-CoV. With more than 118,000 cases reported in 114 countries and nearly 4,291 deaths worldwide, this virus was then labeled as a pandemic by the WHO on 11 March 2020 [5].

COVID-19 has generated significant interest among scientists, researchers, and health care professionals across the globe. Since the emergence of the virus, extensive research is constantly underway to ensure up-to-date information on the manifestation of the virus is present. Relevant information on its mode of transmission, manifestations, pathogenesis, and potential mitigating strategies has started emerging. Although SARS-CoV-2 is primarily a respiratory infection, evidence suggests that patients with a severe infection also present with neurological symptoms such as headache, altered mental status (AMS), hypogeusia, anosmia, and neuralgia. The symptoms may deteriorate and lead to complications such as meningitis, encephalopathy, cerebrovascular events (CVEs), skeletal muscle injury, demyelinating disorders, Guillain-Barre syndrome, and Miller Fisher syndrome [6-7]. However, in the initial stages of the COVID-19 infection, neurological symptoms can be non-specific leading to a gross delay in diagnosis and neurological management. Characteristic symptoms of COVID-19 are isolated to the respiratory system and due to such an association, other symptoms of COVID-19 may be overlooked in the early stages of diagnosis.

We present a comprehensive literature review of all the data and evidence available regarding the neurological manifestations and complications of SARS-CoV-2 infection, to date. The goal of this comprehensive review is to provide a better understanding of the prevalence, severity, progression, and possible outcomes of COVID-19. Furthermore, this review will aim to acclimatize possible outcomes of COVID-19 with, rare but possible invasions of the nervous system in the presence of COVID-19. Being well equipped and updated about possible neurological presentations and complications will not only allow the ability to make a timely diagnosis but also help adopt appropriate management modalities to prevent further complications. Due to the abundance of new researches emerging daily, recently published work, as well as pre-print case reports, small case series, and original papers have been discussed in this review to elucidate the continuum of neurological conditions in SARS-CoV-2 positive cases.

## Review

The pathogenesis of COVID-19 infection on the nervous system can be explained by nervous system invasion and nervous system damage.

Mechanism of nervous system invasion by COVID-19

**Figure 1:**
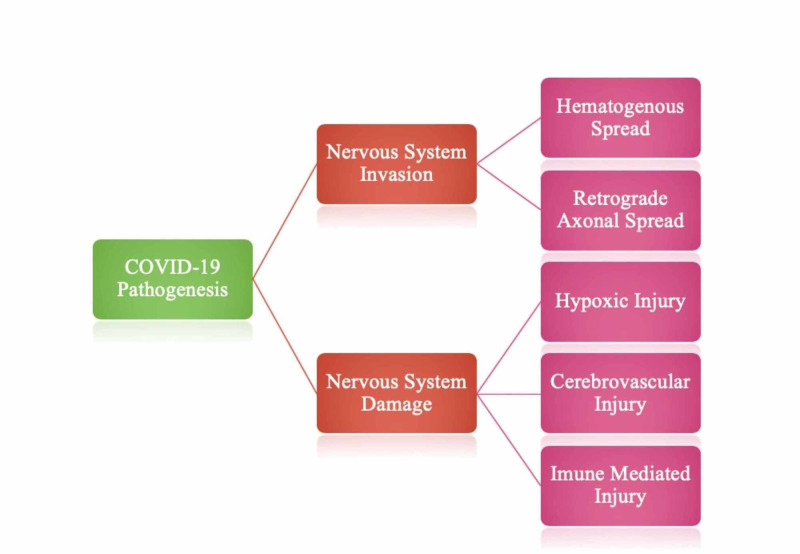
Major modes of nervous system invasion and damage by SARS-CoV-2.

Through various mechanisms that have been suggested in regard to the neurological symptoms prevalent in COVID-19, currently, the most accepted mechanisms of the neurological spread of SARS-CoV-2 are that of hematogenous spread and retrograde axonal spread. Figure 1 depicts the various methods of SARS-CoV-2 spread that have been postulated.

A. Hematogenous Spread

Genetic sequences of the RNA of SARS-COV-2 contain a spike protein sequence that recognizes the ACE2 receptors on the surface of the cellular membrane. ACE2, a metalloproteinase, is present on the surface epithelium of alveoli, intestine, arteries, and veins and acts as functional receptors for SARS-COV-2 [8]. These receptors are not expressed in neurons and microglia hence, the only way to enter the brain is via the vascular network of the brain [9]. The virus first disrupts the nasal epithelium to reach the blood vessels and ultimately reaches the central nervous system (CNS) and other tissues. It is suggested that leukocytes and cytokines, especially interleukin-6 (IL-6), helps in the neural invasion of the virus [10]. This hematogenous spread further supports the CNS involvement through the development of viral encephalitis and myelitis.

B. Retrograde Axonal Spread

Another widely suggested route of invasion is through dissemination via the olfactory nerve. In this route, the virus first invades peripheral neurons and advances towards the CNS system via a mechanism that is referred to as retrograde axonal transport, through synaptic spaces. Although yet to be proven, dissemination into the CNS via olfactory nerve through the cribriform plate has been supported by the presence of the virus in the nasal epithelium, olfactory bulb, and by the development of hyposmia [11]. Thus, the clinicians must perform a thorough workup and consider SAR-COV-2 infection in differentials since hyposmia, anosmia, and ageusia could be the only symptoms in patients with COVID-19 [12]. Moreover, this pathway of CNS involvement remains to be elucidated since olfactory neurons do not express ACE2 receptors and no data has been reported to demonstrate the association between CNS manifestations of COVID-19 and anosmia [13].

Due to the multiple methods of entry of SARS-CoV-2 into the CNS, eradicating the CNS symptoms can prove to be difficult and may pose a serious challenge as clinicians work, undoubtedly, around the clock.

Mechanism of nervous system damage by COVID-19

Neurological damage by SARS-CoV-2 is likely to occur in the three following ways, as shown in Figure 1.

A. Hypoxic Injury

Severe pneumonia secondary to SARS-CoV-2 infection causes respiratory insufficiency and subsequent hypoxia. Chronic systemic hypoxia ultimately causes damage to the brain. The hypoxic state coupled with hypercapnia, peripheral vasodilatation, anaerobic metabolism, and accumulation of toxic metabolites progressively induces cerebral injury due to the development of neural swelling and cerebral edema [14].

B. Immune-mediated Injury

The immune-mediated mechanism of cerebral insult is mainly attributed to the phenomenon, known as the cytokine storm. Cytokine storm involves the overproduction and release of excess amounts of leukocytes and cytokines, especially IL-6 into the blood to help fight infection. Abundant cytokines and leukocytes, in turn, cause increased activation of macrophages, lymphocytes, and endothelial cells leading to over-activation of the complement system and coagulation cascade. Hyperactive immune response, therefore, results in potentially fatal hypercytokinemia and subsequent disseminated intravascular coagulation, ultimately leading to multi-organ failure [15-16].

C. Cerebrovascular Injury

A rise in the luminal pressure of cerebral vessels due to the binding of the virus to ACE2 receptors on blood vessels may also lead to the consequence of intracerebral hemorrhage [17]. Dysfunction in the coagulation system, in cases such as thrombocytopenia, and increased D-dimer levels are noted as other high-risk factors for intracranial hemorrhage that have been frequently seen in severely ill patients with SARS-CoV-2 infection [18].

Neurological manifestations of COVID-19

Patients with SARS-CoV-2 may have symptoms of varying degrees, ranging from a mild cough/fever to multiple organ dysfunction that can progress to multiple organ system failures. Clinical data has currently demonstrated that some COVID-19 patients may also have symptoms similar to that of intracranial infections such as headache, dizziness, altered mental status (AMS), and epilepsy. Also, an increasing number of COVID-19 patients experience a sudden loss of odor or taste sensation indicating a peripheral nervous system (PNS) invasion. Therefore, olfactory and taste disorders (OTD) can also be observed in COVID-19 [19-21].

In a retrospective, observational case series, from Wuhan, China, Mao et al. evaluated 214 COVID-19 positive patients (mean age = 52.7 years) hospitalized in Union Hospital of Huazhong University of Science and Technology in Wuhan, China [6]. Results showed that overall, 36.4% of patients (n = 78) had neurologic manifestations. The neurological manifestations were described into three categories: (1) CNS manifestations, (2) PNS manifestations, and (3) skeletal muscle injury manifestations.

**Figure 2:**
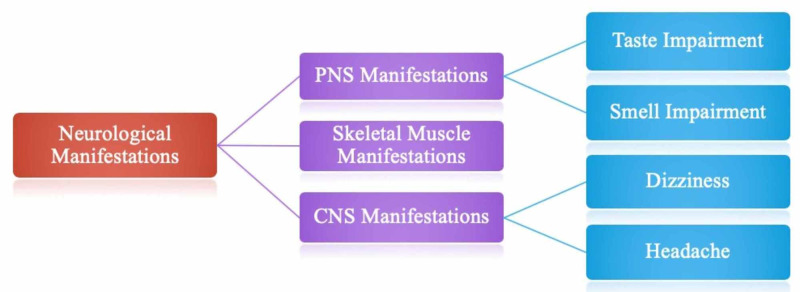
Categorization of neurological manifestations and most reported symptoms.

According to the results by Mao et al., the most commonly reported CNS symptoms were dizziness (n = 36 [16.8%]) and headache (n = 28 [13.1%]); whereas the most frequently reported PNS symptoms were taste (n = 12 [5.6%]) and smell (n = 11 [5.1%]) impairment [22]. These findings have been presented in a diagrammatic format in Figure 2. However, it should be noted that whether non-specific neurological symptoms such as headache, dizziness, depressed consciousness, or seizures are consequences of the disease itself needs to be further investigated [6].

In a single-center prospective case series, Helms et al. investigated neurological features of 58 COVID-19 positive patients admitted in Strasbourg University Hospital, France. Neurological manifestations were recorded in 49 out of 58 (84%) patients with COVID-19-related acute respiratory distress syndrome (ARD) including encephalopathy, notable confusion and agitation, and corticospinal tract signs such as ankle clonus, hyperreflexia, and Babinski reflex. As assessed by the confusion assessment method for the intensive care unit (CAM-ICU) scale, agitation was the most common symptom occurring in 69% of hospitalized patients (n = 40) followed by confusion in 26/40 patients (65%). Corticospinal tract signs (n = 39, 67%) were the second most common neurological manifestation in these patients. The study also reported a dysexecutive syndrome in 14 out of 39 patients at discharge which encompasses behavioral and cognitive features like poor attention span, disorientation, and poorly defined command-response gestures [7].

Yin et al., retrospectively, evaluated clinical features of 106 COVID-19 positive patients with neurological manifestations in Huoshenshan Hospital in Wuhan, China. The patients were divided into two groups of severe and non-severe disease. Fever (n = 62, 58.5%) was found to be the most common symptom. Among neurological symptoms, myalgia (n = 26, 24.5%) was the leading symptom. Myalgia was followed by paralysis of extremities (n = 20, 18.9%), depressed consciousness (n = 17, 16%), and positive focal neurological signs (n = 42, 39.6%) in occurrence. The study also revealed that patients in the severe group were likely to have AMS [22]. A high incidence of AMS may be attributed to deranged toxic metabolites, adverse effects of medications, or primary nervous dysfunction in severely diseased patients.

Post-viral loss of sense of smell is one of the leading causes of anosmia in adults, accounting for up to 40% anosmia cases. Mechanism of post-viral anosmia is poorly understood; however, it is plausible that infection of olfactory bulb via direct invasion through olfactory neurons plays a key role [23]. In addition, an experiment conducted on mice also demonstrated the transneuronal spread of previous SARS-CoV infection via olfactory bulb [24]. It is, therefore, no surprise that SARS-CoV-2 would also cause anosmia in infected individuals. In March 2020, a team from King’s College, London added loss of sense of smell as one of the symptoms of COVID-19. In a research update released on April 1, 2020, it was stated that impaired sense of taste or smell may be a much stronger determinant of SARS-Cov-2 infection than fever [21].

In a cross-sectional survey, Giacomelli et al. interviewed 59 patients of COVID-19 admitted to L. Sacco Hospital in Milan, Italy. Of these, 20 (33.9%) patients reported either taste (dysgeusia or ageusia) or olfactory disorders (hyposmia or anosmia) [19]. Eleven (18.6%) patients reported both. Twelve (20.3%) patients experienced the symptoms before hospitalization, whereas eight (13.5%) patients developed the symptoms during their stay at the hospital. The authors also noted that changes in taste were more common (91%) prior to hospital admission, while taste and smell alternations were reported equally frequently after hospitalization. Females (52.6%) more frequently reported a lack of taste or smell in comparison to males (25%) [19]. Moreover, patients with at least one OTD were younger than those without; currently, there is no exact pathogenesis present for why OTD may be more prevalent in younger patients.

Bagheri et al. reported the results of an Iranian cohort of COVID-19 patients as participants. The results stated that anosmia and hyposmia was a symptom in 48.3% of the responses, while 83.38% of responses reported a reduced taste sensation [20]. 76.24% of the patients reported that the anosmia was acute in occurrence. Other features reported before the onset of anosmia ranged from flu-like symptoms (75.5%), headaches (48.6%), and nasal stiffness (43.7%) to fever (37.3%). Furthermore, the results also revealed that anosmic patients were more likely to experience dysgeusia. It was also noted that the majority of these patients lacked typical symptoms such as cough and fever.

Another cross-sectional study supporting the smell and taste dysfunction has been done by Yan et al. [25]. This study was done on COVID patients in a single-center from the USA. A loss of smell was seen in 68%, and taste impairment in 71% of the COVID-19 positive patients was reported. The authors of this study hypothesized that since the patient population under consideration was ambulatory, the route of infection might be nasal in such patients relative to a pulmonary spread in critically ill patients.

Table 1 summarizes the studies discussed above.

**Table 1:** Summary of reported cases of central nervous system (CNS) complications of COVID-19.

	Mao et al. [6]	Helms et al. [7]	Yin et al. [22]	Giacomelli et al. [19]	Bagheri et al. [20]	Yan et al. [25]
Study design	Retrospective	Prospective	Retrospective	Cross-sectional	Cross-sectional	Cross-sectional
Number of patients	214	58	106	59	10069	59
Mean age (years)	52.7	63	72	60	32.5	45
Headache	13.1%	-	7.5%	3.4%	-	66.1%
Dizziness	18.8%	-	8.5%	-	-	
Impaired consciousness	14.8%	-	16%	-	-	
Hyposmia	5.1%	-	-	5.1%	76.2% (sudden) 60.9% (progressive)	68%
Hypogeusia	5.6%	-	-	10.2%		71%
Both hyposmia and hypogeusia	-	-	-	18.6%	83.3%	
Skeletal muscle injury/myalgia	19.3%	-	24.5%	-	-	63%
Agitation	-	69%	-	-	-	-
Confusion	-	65%	-	-	-	-
Corticospinal tract sign	-	67%	-	-	-	-

Central nervous system complications of COVID-19

**Figure 3:**
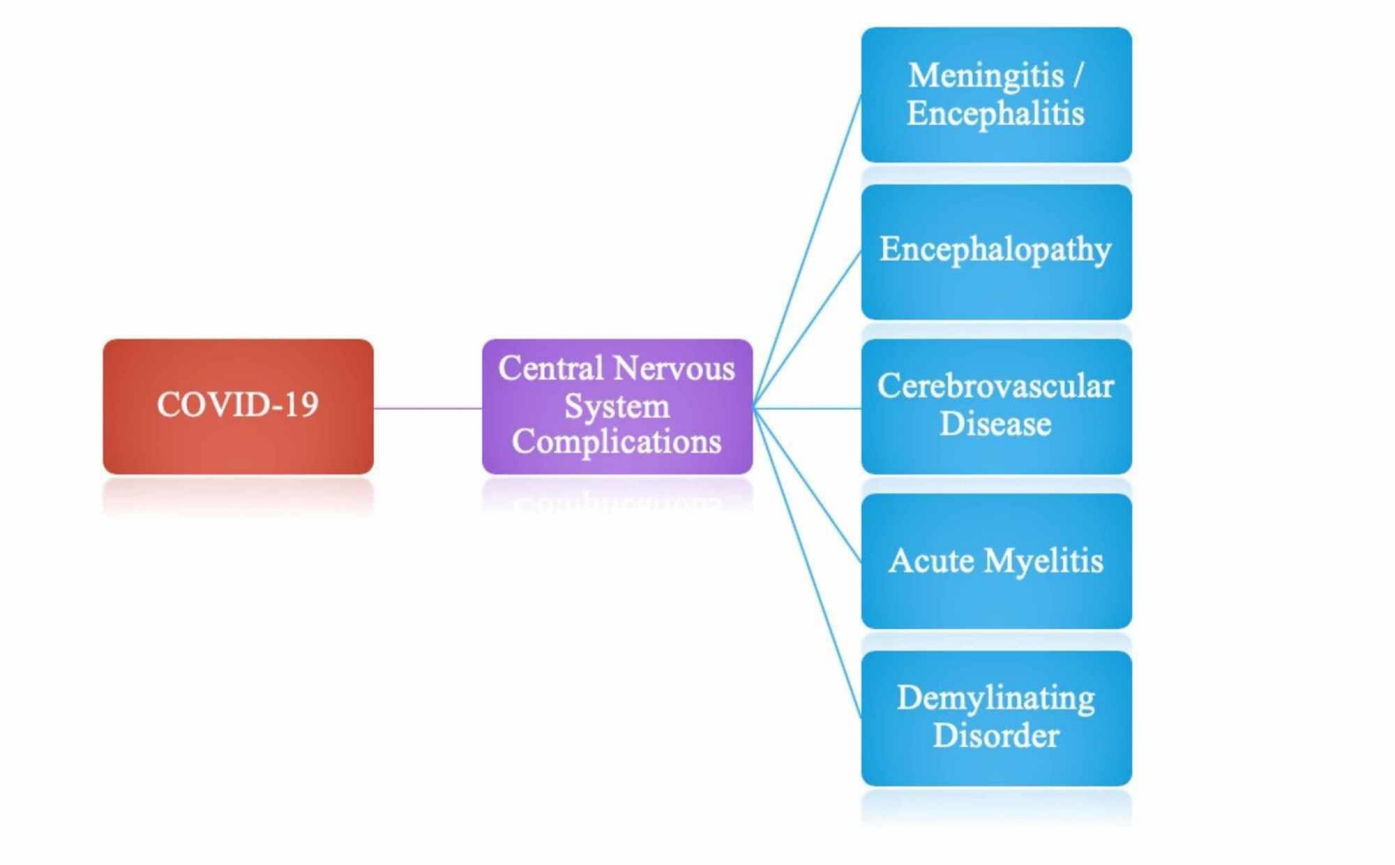
Central nervous system manifestations of COVID-19.

Figure 3 is a summary figure that denotes all of the central nervous system complications that have been linked to COVID-19. Each of the listed complications will be discussed in further detail, however, extensive research has revealed these complications as the most notable CNS manifestations of COVID-19.

A. Meningitis and Encephalitis

As per currently available research, at least three case reports of SARS-CoV-2 encephalitis have been reported [26-28]. The first case was reported in Beijing Ditan Hospital by Xiang et al. when a patient presented with repeated maxillofacial and angular twitching a few weeks after disease onset [26]. Diminished response to light, increased muscle tension in extremities, neck resistance and bilateral ankle clonus were evident on physical exam. Positive SARS-CoV-2 RNA testing of cerebrospinal fluid (CSF) led to the diagnosis of encephalitis.

Moriguchi et al. shared their experience in dealing with a 24-year-old man who presented with sudden onset convulsions with obvious neck stiffness followed by loss of consciousness and a one-day history of fever [27]. He had no history of traveling or contact with a COVID-19 positive individual. Although nasopharyngeal swab was tested negative for SARS-CoV-2, the viral presence was confirmed in the CSF thereby confirming the first reported case of COVID-19 caused meningitis/encephalitis. Magnetic resonance imaging (MRI) of the brain showed hyperintense signals along the inferior lobe of the right lateral ventricle. Hyperintensity was also noted in the right mesial temporal lobe and hippocampus. The abnormal MRI findings were suggestive of encephalitis and hippocampal sclerosis. Hippocampal sclerosis was ruled out because the patient had no past history of mesial temporal epilepsy. The authors, thus, concluded that it was a case of SARS-CoV-2 meningitis/encephalitis.

Ye et al. also reported a case of SARS-CoV-2 infection-related encephalitis. A Wuhan male patient presented with an altered level of consciousness which progressed to confusion [28]. He had a history of fever, shortness of breath, and myalgia for 10 days. A computed tomography (CT) scan of the brain was normal, whereas the CT scan of the chest showed multiple ground-glass opacities. On physical examination, nuchal rigidity, Kernig's sign, and Brudzinski's sign along with extensor plantar response were present indicating meningeal irritation. CSF was further tested negative for SARS-CoV-2. After careful evaluation by neurologists, the corroborative diagnosis of coronavirus-associated encephalitis was established.

B. Encephalopathy

Mao et al. reported headache and encephalopathy in 40% of patients in their cohort but the details and the diagnostic criteria used were not described [6]. In a report by Filatov et al., a 74-year-old man who had a travel history from Europe to the United States presented with fever, cough, and AMS [29]. CSF examination for SARS-CoV-2 and skull CT was negative for infection. The diagnosis for COVID-19-associated encephalopathy based on electroencephalogram (EEG) findings was consistent with encephalopathy, focal left temporal lobe dysfunction, and possible epileptogenicity.

Another very recent report from Michigan, United States, describes the first reported case of acute hemorrhagic necrotizing encephalopathy (AHNE) in COVID-19 patients [30]. A female patient in her fifties presented with AMS, cough, and fever. She was diagnosed with AHNE based on imaging findings. Non-contrast CT imaging of the brain showed bilateral symmetric hypo-attenuation within the medial thalami and MRI showed hemorrhagic ring-enhancing lesions within the bilateral thalami, medial temporal lobes, and sub insular regions. The proposed mechanism is likely due to cytokine storm which disrupts the blood-brain barrier and damage to the brain parenchyma.

C. Cerebrovascular Disease

Another striking observation is the prevalence of COVID-19-associated cerebrovascular events (CVE). A significant body of evidence indicates that respiratory infection, in particular, is an independent risk factor for acute cerebrovascular disease [31]. A retrospective case series study from Wuhan, China reported six cases of CVE in their cohort of 214 patients, whereas a French cohort had three cases of ischemic stroke which were detected on neuroimaging when patients underwent imaging for encephalopathy [6-7].

Sharifi et al. reported a case of intracerebral hemorrhage in a 79-year-old COVID-19 positive Iranian male [32]. The patient presented to an emergency department due to an acute loss of consciousness with a history of cough and fever for three days. He had no history of hypertension or anticoagulation. Lung CT showed ground-glass appearance whereas brain CT revealed intracerebral hemorrhage in the right cerebrum along with subarachnoid and intraventricular bleeding. The patient was later diagnosed as a case of COVID-19 upon doing a polymerase chain reaction (PCR) of an oropharyngeal swab. Sharifi et al. hypothesized that the involvement of brain ACE2 disrupted normal cranial blood flow resulting in arterial wall rupture. This evidence justifies the involvement of ACE2 in the pathogenesis of viral invasion of the brain and suggests that infection with SARS-CoV-2 should be considered when dealing with a patient presenting with acute intracranial hemorrhage.

D. Acute Myelitis

In a case reported by Zhao et al., a 66-year-old male presented with bilateral flaccid paralysis coupled with urinary and bowel incontinence [33]. Following his admission, his PCR was positive for COVID-19. Imaging revealed patchy high density and ground glass shadow in his right lung. Bilateral basal ganglia and periventricular infarction were also observed. He presented with spinal cord involvement one week after the fever, which the authors describe as acute post-infectious myelitis. This is the first study that reports such a complication. Previously, acute myelitis had always been known to occur by infectious microbes like Epstein-Barr virus (EBV), cytomegalovirus (CMV), and Mycoplasma pneumonia among others. Zhao et al. suggest that this complication may have been caused by a cytokine storm, considering the elevated levels of interleukin-6, C-reactive protein, and serum ferritin. However, the lack of spinal imaging may have been a possible limitation in this case. Nevertheless, it is essential to keep acute myelitis in mind when diagnosing a COVID-19 patient with a similar presentation.

E. Demyelinating Disorder

Apart from encephalitis and toxic encephalopathy, demyelinating disorders of CNS following a viral infection are also common. Recently, Zanin et al. reported a case of a 54-year-old woman having demyelinating lesions in the brain and spine induced by SARS-CoV-2 [34]. She was found unconscious at home and experienced generalized seizures upon regaining consciousness.

She also had anosmia and ageusia for the past few days and a history of anterior communicating artery aneurysm which was treated 20 years back. Her head CT was normal; chest X-ray revealed interstitial pneumonia and PCR for SARS-COV-2 came positive. EEG showed two seizures and MRI of the brain revealed alterations of the periventricular white matter, hyperintense lesions in T2-weighted imaging (T2WI), without the restriction of diffusion nor contrast enhancement. Similar lesions were found in the cervical and dorsal spinal cord at the bulbo-medullary junction. Surprisingly, CSF PCR for SARS-COV-2 was negative. Other conditions such as multiple sclerosis, viral and bacterial infection were ruled out. This case suggests that the pathogenesis of viral infection was due to systemic inflammatory response syndrome (SIRS). The pro-inflammatory condition caused by virus-induced cytokine storm seems to be responsible for glial cell activation and subsequent demyelination [15]. Production of antibodies against glial cells triggered by the virus in the post-infective period was another possibility that was speculated.

Table 2 provides a summary of all the case reports discussed above.

**Table 2:** Summary of reported cases of central nervous system (CNS) complications of COVID-19.

AUTHOR	LOCATION	AGE, GENDER	PRESENTATION AND DIAGNOSIS	RADIOLOGICAL FINDINGS
Xiang et al. [26]	Beijing, China	-	PRESENTATION: frequent maxillofacial and angular twitching with persistent hiccups at 2 weeks after disease onset. DIAGNOSIS: SARS-CoV-2 associated encephalitis	-
Zhao et al. [33]	Wuhan, China	66 years, male	PRESENTATION: Flaccid paralysis of bilateral lower limbs and urinary and bowel incontinence. DIAGNOSIS: Post-infectious acute myelitis related to COVID-19 infection.	CHEST CT: Patchy high-density blurred shadow in the upper lobe of the left lung and patchy ground-glass shadow in the anterior segment of the upper lobe of the right lung. CRANIAL CT: Bilateral basal ganglia and paraventricular lacunar infarction, brain atrophy.
Filatov et al. [29]	Boca Raton, USA	74 years, male	PRESENTATION: Fever, cough, and altered mental status. DIAGNOSIS: COVID-19-associated encephalopathy	CHEST X-RAY: Bilateral ground-glass opacities with evidence of effusion. CHEST CT: Patchy bibasilar consolidations and subpleural opacities.
Moriguchi et al. [27]	Yamanashi, Japan	24 years, male	PRESENTATION: Convulsions with loss of consciousness. DIAGNOSIS: aseptic encephalitis with SARS-CoV-2 RNA in cerebrospinal fluid.	BRAIN MRI: Diffusion-weighted imaging (DWI) showed hyperintensity along the wall of the inferior horn of the right lateral ventricle. FLAIR images showed hyperintense signal changes in the right mesial temporal lobe and hippocampus with slight hippocampal atrophy. These findings indicated right lateral ventriculitis and encephalitis mainly on the right mesial lobe and hippocampus. T2W image showed pan-paranasal sinusitis.
Sharifi et al. [32]	Sari, Iran	79 years, male	PRESENTATION: Acute loss of consciousness. DIAGNOSIS: Intracranial bleed associated with SARS-CoV-2.	CHEST CT: Ground-glass appearance. BRAIN CT: Intra-cerebral hemorrhage in the right cerebrum along with subarachnoid and intra-ventricular bleeding.
Poyiadji et al. [30]	Michigan, USA	50 years, female	PRESENTATION: Cough, fever, and altered mental status. DIAGNOSIS: Acute hemorrhagic necrotizing encephalopathy	HEAD CT WITHOUT CONTRAST: Symmetric hypo attenuation within the bilateral medial thalami. BRAIN MRI: Hemorrhagic rim enhancing lesions within the bilateral thalami, medial temporal lobes, and sub-insular regions.
Ye et al. [28]	Wuhan, China	male	PRESENTATION: Altered level of consciousness which progressed to confusion. DIAGNOSIS: SARS-CoV-2 infection-related encephalitis.	CHEST CT: Multiple ground-glass opacities
Zanin et al. [34]	Brescia, Italy	54 years, female	PRESENTATION: Unconsciousness, seizures, anosmia, and ageusia. DIAGNOSIS: SARS-CoV-2 induced brain and spine demyelinating lesions.	CHEST CT: Revealed interstitial pneumonia. BRAIN MRI: T2WI images showed hyperintense lesions. Similar lesions were found in the cervical and dorsal spinal cord at bulbo-medullary junction.

Peripheral nervous system complications of COVID-19

**Figure 4:**
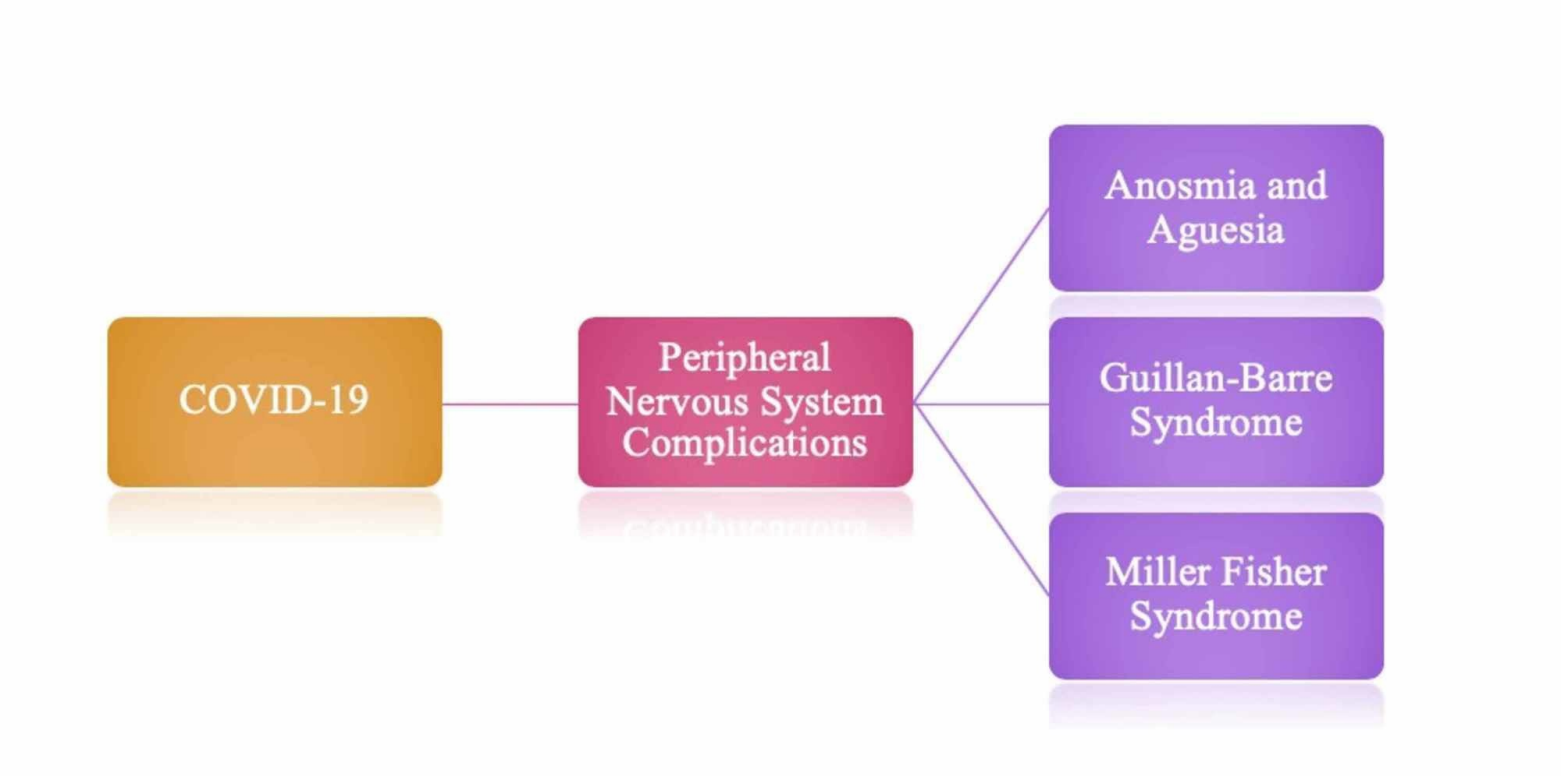
Peripheral nervous system manifestations of COVID-19.

Figure 4 denotes the peripheral nervous system complications of COVID-19. As done with the central nervous system complications, the complications mentioned in Figure 4 will be further discussed in detail.

A. Anosmia and Ageusia

Anosmia and ageusia are common neurological findings in patients with COVID-19 as described above. Mao et al. reported taste impairment in 5.6% and smell impairment in 5.1% patients from a Chinese cohort [6]. Various literature from case reports also supports this finding. Hjelmesæth and Skaare describe a case with the loss of smell and taste as the only presenting symptom of COVID-19 [35]. In this case, three related patients, with ages ranging from 60 to 90 years, experienced symptoms such as anosmia, ageusia, and dysgeusia. All three of the individuals later tested positive for SARS-CoV-2 RNA. Only one of them mentioned that the individuals developed fever and cough along with the dysgeusia while the others had no additional symptoms. The authors hypothesize that this might be due to the expression of the ACE2 receptor on the oral mucosa, especially on the tongue epithelium [34-35].

In another case report, an African American female presented with distorted taste sensation and a diminished sense of smell which eventually progressed to complete anosmia [36]. She later developed a cough; chest tightness and physical exam were positive for bilateral wheezes and rales. Imaging showed multiple ground-glass lung opacities. After confirmation of the SARS-CoV-2 infection, the patient was treated with hydroxychloroquine and azithromycin. Following her treatment, the patient regained both her senses and recovered uneventfully.

Another report describes a physician who developed mild flu-like symptoms with a sudden onset of persistent anosmia and ageusia. Sniffin’ Sticks test was used to verify anosmia while the ageusia was tested with taste screening among other tests [37].

B. Guillain-Barre Syndrome

Guillain-Barre syndrome (GBS) is an acute immune-mediated disease of the peripheral nerves and nerve roots. It is associated with various infectious agents and presents with ascending flaccid paralysis with areflexia/hyporeflexia spread over a course of days to weeks. It can often involve cranial nerves, with the involvement of the facial nerve being the most common [38]. An association of GBS with COVID-19 was first reported by Zhao et al. in a 61-year-old female who presented with severe fatigue and acute lower extremity weakness [39]. She had a travel history to Wuhan, China. Nasopharyngeal PCR was positive for COVID-19. The diagnosis of COVID-19-associated GBS was based on areflexia. Nerve conduction studies (NCS) and electromyography (EMG) findings were consistent with findings present in GBS. Sedaghat and Karimi reported an elderly male 65-year of age, that presented with progressive weakness of his limbs leading to quadriplegia before admission [40]. He also had bilateral facial paralysis, cough, dyspnea, and fever. He was tested positive for COVID-19 soon after admission. Absent deep tendon reflexes, bilateral ground-glass lung opacities, and electromyography findings confirmed the diagnosis of GBS. The exact mechanism of GBS in COVID-19 infection is unknown; however, the authors consider that the inflammatory cytokines might have a role in this, as these cytokines produce an immune reaction [41].

Toscano et al. also reported the incidence of neurologic symptoms specifically pointing towards GBS in five COVID-19 patients [42]. These symptoms included weakness and paresthesias of the limbs, facial diplegia, and ataxia. These symptoms progressed to flaccid tetraparesis or tetraplegia with time. The diagnosis of the axonal variant of GBS in three patients and the demyelinating process in two patients was based on fibrillation potentials on electromyography. Virani et al. reported similar findings in a patient that presented with lower extremity weakness and was later diagnosed with COVID-19 infection by a nasopharyngeal PCR [43]. This patient had a history of Clostridium difficile colitis. The infection history, ascending paralysis and physical exam findings were all, collectively, consistent with the diagnosis of GBS.

C. Miller Fisher Syndrome

Miller Fisher syndrome (MFS) presents with acute onset external ophthalmoplegia, ataxia coupled with the loss of tendon reflexes. Usually, MFS occurs following a viral illness similar to GBS. The most common infectious agents are Haemophilus influenzae, Campylobacter jejuni, and CMV [44]. Gutiérrez-Ortiz et al. described to the best of their knowledge, the first case of MFS and isolated multiple cranial neuropathies in association with SARS-CoV-2 infection [45]. Their article summarizes two case reports.

The first patient is a 50-year-old male who presented with complaints of impaired sense of smell and taste, areflexia, ataxia, right fascicular oculomotor nerve palsy, right internuclear ophthalmoparesis, albuminocytologic dissociation and positive testing for GD1b-IgG antibodies. With an oropharyngeal swab positive for SARS-CoV-2, his treatment was soon started with intravenous immunoglobulin. Soon after treatment, all of his neurological symptoms resolved besides anosmia and ageusia. The second patient, a 39-year-old male, presented with ageusia, areflexia, bilateral abducens palsy, and albuminocytologic dissociation. This was preceded a few days before with diarrhea and low-grade fever. With a positive oropharyngeal swab for COVID-19, this patient was treated with acetaminophen and showed complete neurological recovery. His diagnosis was polyneuritis cranialis, often called isolated multiple cranial neuropathies.

Though the occurrence of MFS and polyneuritis cranialis might be less likely, it is pivotal to consider these complications in a COVID-19 patient with a similar presentation.

Table 3 provides a summary of all the case reports discussed above.

**Table 3:** Summary of reported cases of peripheral nervous system (PNS) complications of COVID-19. *Only abstract is available in English.

AUTHOR	LOCATION	AGE, GENDER	PRESENTATION AND DIAGNOSIS	RADIOLOGICAL FINDINGS
Haldrup et al.* [37]	Denmark	30 years, -	Presentation: Mild flu and sudden anosmia and ageusia	-
Zhao et al. [39]	Jingzhou, China	66 years, Female	Presentation: Weakness in both legs, fatigue. Diagnosis: Guillain-Barre syndrome	-
Hjelmesæth and Skaare [35]	Oslo, Norway	1. In 60 years, female 2. In 60 years, male 3. In 90 years, male	Presentation: 1. Anosmia, ageusia. 2. Anosmia, ageusia. 3. Anosmia, dysgeusia, cough, dyspnea, and fever	-
Sedaghat and Karimi [40]	Sari, Iran	65 years, male	Presentation: Acute progressive symmetric ascending quadriparesis. Diagnoses: Guillain-Barre syndrome	Lung CT showed diffused consolidations and ground-glass opacities in both lungs and bilateral pleural effusion.
Gutiérrez-Ortiz et al. [45]	Madrid, Spain	1. 50 years, male 2. 39 years, male	Presentation: 1. Ageusia, right internuclear ophthalmoparesis, right fascicular oculomotor palsy, ataxia, areflexia. 2. Ageusia, bilateral abducens palsy, areflexia, and albuminocytologic dissociation. A few days before, he had developed diarrhea and a low-grade fever. Diagnosis: Miller Fisher syndrome and polyneuritis cranialis in COVID-19 patients.	Chest X-ray and head CT without contrast were normal for both patients.
Melley et al. [36]	Pennsylvania, USA	59 years, female	Presentation: Disturbed taste and a reduced sense of smell which progressed to anosmia	Chest X-ray and CT chest: multiple patchy ground-glass opacities in bilateral subpleural areas

Skeletal muscle injury

**Figure 5:**
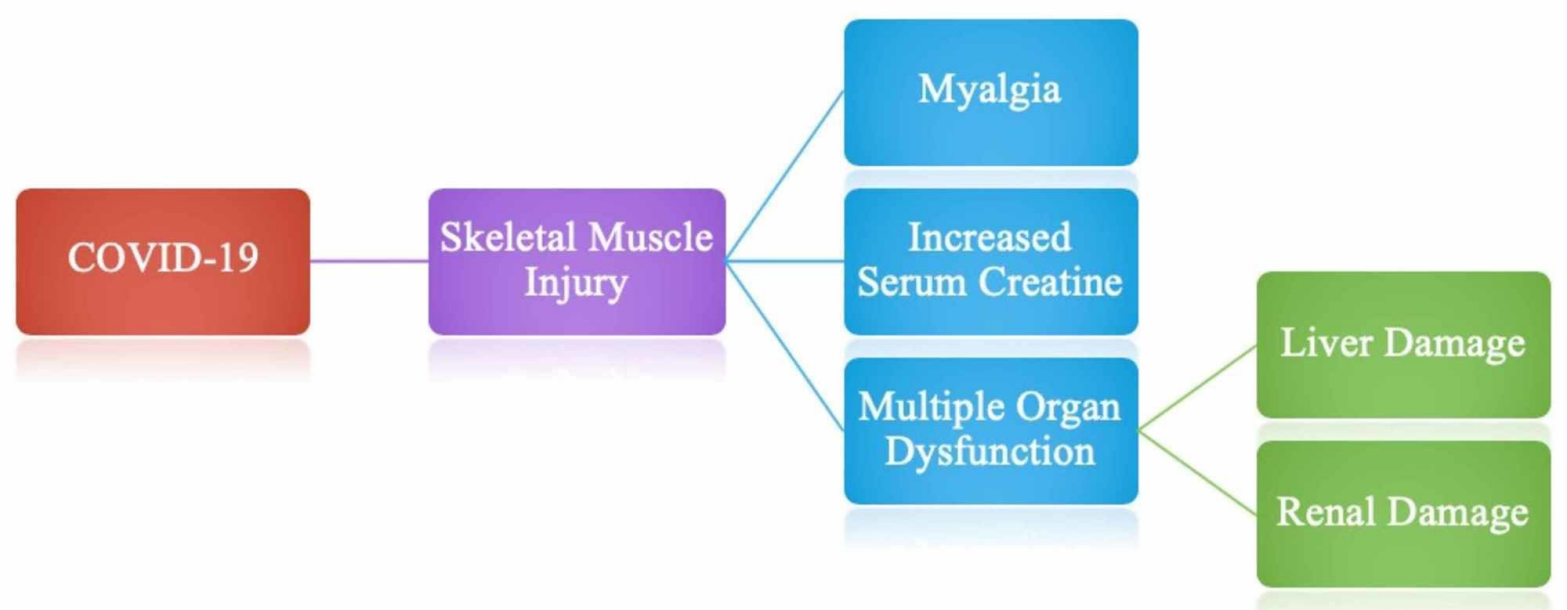
Skeletal muscle injury complications associated with COVID-19.

Muscle injury is defined as a patient having myalgia and increased serum creatine kinase levels above 200 U/L [46]. As depicted in Figure 5, muscle injury in SARS-COV-2 patients is often associated with multiple organ dysfunctions. Mao et al. reported that out of 214 patients, 23 (10.7%) had muscle injury of which 17 (19.3%) were severely ill and six (4.8%) were in the non-severe category [6]. It was noted that in severely ill patients, there was an increased inflammatory response (decreased lymphocytes and increased C-reactive protein) with more muscle damage (increased creatine kinase levels), liver damage (increased aspartate aminotransferase, alanine aminotransferase, and lactate dehydrogenase levels) and kidney damage (increased creatinine and blood urea nitrogen levels). Moreover, it is speculated that patients with pre-existing comorbidities are more prone to develop muscle injury. Although Figure 5 only denotes liver and renal damage in association with a skeletal muscle injury, it is imperative to mention that other organs may also be damaged, however, further research in this association is necessary.

The exact mechanism underlying muscle injury in SARS-COV-2 patients is yet to be known, however, it is hypothesized that expression of ACE2 receptors on skeletal muscles and viral invasion through these functional receptors play a key role in the pathogenesis of injury [47]. Another proposed mechanism suggests that the phenomenon of muscle injury is the result of infection mediated immune response causing a surge of inflammatory cytokines and immune mediators in the blood serum that leads to muscle fiber damage. Further studies are required to shed some light on mechanisms giving rise to skeletal muscle damage in SARS-COV-2 patients.

Association of neurological manifestations of COVID-19 with pre-existing comorbidities

Another area of concern is the increased susceptibility of patients with pre-existing comorbidities to develop COVID-19-associated neurological manifestations. Data from various studies suggest that patients with underlying disorders are more likely to have a severe infection and thus, are more prone to developing neurological symptoms and complications.

Mao et al. found that neurological symptoms were more frequent in severe disease patients (45.5% vs 30.2%) [6]. The research described severity based on respiratory characteristics, defining mild as not necessitating respiratory support and severe as requiring ventilation. The more dramatic neurological symptoms were observed in older patients with pre-existing medical comorbidities such as hypertension (n = 51), diabetes (n = 30), cardiac or cerebrovascular disease (n = 15), and malignancy (n = 13). This indicates that patients with comorbidities are more susceptible to neurological manifestations in the disease course of COVID-19.

To further elucidate the effect of comorbidities on the severity of SARS-CoV-2 infection, Yang et al. carried out a meta-analysis to investigate the prevalence of comorbidities in the COVID-19 patients and its correlation with severity of the disease. The results showed that hypertension and diabetes were the most prevalent comorbidities, followed by cardiovascular and respiratory diseases [48]. The pooled odds ratio of hypertension, respiratory, and cardiovascular diseases was 2.36, 2.46, and 3.42, respectively, when compared between severe and non-severe patients. Authors, therefore, concluded that underlying diseases may be risk factors for a more severe clinical course.

Furthermore, patients with pre-existing neurological comorbidities also tend to develop neurological manifestations as supported by a retrospective case series of 106 COVID-19 positive patients with comorbid neurological conditions; 74.4% had a previous cerebral infarction, 18.9% had dementia, 9.4% had acute cerebral infarction, five had followed by sequelae of a cerebral hemorrhage in 4.7%, intracranial mass lesions in 3.8%, epilepsy in 2.8%, Parkinson’s disease in 1.9%, myelopathy in 0.9% [22]. The evidence stated above indicates that comorbidities may be linked to the development of neurological manifestation. Thus, clinicians must pay importance to the comorbid conditions when evaluating the risk margin of a COVID-19 patient.

Neurologists' viewpoint

From a neurologist's perspective considering the rapid patient influx, usual non-emergency procedures have been rescheduled like elective epilepsy monitoring to make sure that beds are vacant for COVID-19 patients.

Another newly emerging dimension of medicine is the telehealth systems. With the outdoors having an increased risk of viral transmission, many patients have been directed towards online consultations. Teleneurology has made sure that the patients are facilitated in every way possible. Efforts to limit doctor-patient interaction are made to reduce viral transmission. Doctors have been trying to reduce the number of elective diagnostic and surgical procedures unless necessary. To limit exposure, medical students are being taught through online classes and are being provided with online study material.

For a neurologist in such times, it is necessary to be open-minded when treating a COVID-19 patient. Neurologists are expected to thoroughly evaluate the patient’s presenting features and compliment them with radiographic findings and cerebrospinal fluid analysis. Differentiating the direct effect of coronavirus against the systemic effects (sepsis, hypoxia, and hypercoagulable states) of the virus on the nervous system is also encouraged. Neurologists need to be careful and take all the necessary precautions for the safety of their patients as well as themselves. Both the neurologists and patients must don protective disposable masks, latex gloves, caps, and carry sanitizers containing ethanol or hydrogen peroxide. All patients as well as doctors must regularly have their temperature checked before they enter the consultation room. It is imperative that proper counseling regarding sanitary techniques to limit viral transmission is done for every patient [5-7].

Adopting a more futuristic approach may prevent long-term damage [5-7]. Bearing in mind all the known neurological complications of coronavirus and its mechanism, neurologists should formulate a strategy for preventing and limiting the progression of the disease. This, however, demands neurologists and other healthcare professionals to work in collaboration to ease the burden and work effectively. In order to treat those with severe infection admitted in ICU, we recommend that a team of healthcare providers should be set up to look out for each other. If one of them falls, others will be able to take over and help treat the patient. In addition, patients with pre-existing neurological disorders, particularly those on immunosuppressants, will warrant close monitoring. Thereby, neurologists will have to be more active in the frontline and be extra vigilant about the neurological consequences of COVID-19.

## Conclusions

Given the global dimension of the COVID-19 and rising number of fatalities every day, it is essential that this issue is tackled through a multidisciplinary clinical and research perspective. As the impact of SARS-CoV-2 on the nervous system increases, neurologists face a very real challenge in terms of attempting to note the early onset of neurological symptoms. This review summarizes the available information in a concise and more absorbable manner so readers may benefit from it. The review states that the coronavirus may alter the neuronal mechanism either directly, or may exaggerate a preexisting condition. Knowing that this viral infection can result in long-term damage, we propose that patients should be followed up by careful imaging and laboratory as well as neurological assessment to determine the magnitude of brain damage.
